# Advances in molecular interactions between rice and insect herbivores

**DOI:** 10.1007/s44297-024-00027-y

**Published:** 2024-05-01

**Authors:** Peng Kuai, Yonggen Lou

**Affiliations:** 1 State Key Laboratory of Rice Breeding and Biology & Ministry of Agriculture Key Lab of Molecular Biology of Crop Pathogens and Insects, Key Laboratory of Biology of Crop Pathogens and Insects of Zhejiang Province, Institute of Insect Sciences, Zhejiang University, Hangzhou, 310058 China; 2 Hainan Institute, Zhejiang University, Sanya, 572025 China

**Keywords:** Rice, Immune receptors, Herbivore-associated molecular patterns, Damage-associated molecular patterns, Effectors, Herbivore-induced plant defenses

## Abstract

To adapt to each other, plants and insect herbivores have developed sophisticated molecular interactions. Here, we summarize current knowledge about such molecular interactions between rice, a globally important food crop, and insect herbivores. When infested by insect herbivores, rice perceives herbivore- and/or damage-associated molecular patterns (HAMPs/DAMPs) via receptors that activate early signaling events such as the influx of Ca^2+^, the burst of reactive oxygen species, and the activation of MPK cascades. These changes result in specific rice defenses via signaling networks that mainly include phytohormones (jasmonic acid, salicylic acid, ethylene, and abscisic acid) and transcription factors. Some compounds, including flavonoids, phenolamides, defensive proteins, and herbivore-induced rice volatiles, have been reported to be used by rice against insects. Insect herbivores can deliver effectors or factors to inhibit rice defenses or enhance rice susceptibility. Although the number of HAMPs and defense-suppressing effectors from rice piercing-sucking insects has increased rapidly, none from rice chewing insects has been identified. Moreover, herbivore effectors or factors that induce rice susceptibility, and rice immune receptors recognizing HAMPs or effectors, are not well characterized. We point out future research directions in this area and highlight the importance of elucidating the mechanisms for rice sensing of insect herbivores and for insect counter-defenses against plants.

## Introduction

Over millions of years of coevolution, plants and herbivorous insects have engaged in an ongoing battle involving diverse mechanisms. Plants employ constitutive defenses to prevent herbivore infestation; when attacked by insect herbivores, plants activate induced defenses by perceiving herbivore- and/or damage-associated molecular patterns (HAMPs/DAMPs) [1, 2]. These induced defenses are produced locally and systemically, and may influence the performance of conspecific and non-conspecific herbivores that share the same host plant, either simultaneously or successively, directly and/or indirectly, by attracting the natural enemies of insect herbivores [3–5]. In response to plants, insect herbivores have evolved various counter-defense strategies, such as secreting effectors or factors to inhibit plant defenses or enhance plant susceptibility, and detoxifying or accommodating plant defensive compounds [4, 6, 7]. These molecular interactions between plants and insect herbivores largely determine the consequences of plant-herbivore interactions that are observable at the macroscopic level: the plant either resists or is susceptible to the herbivore, which in turn affects the population density and diversity of both plants and insect herbivores. Therefore, elucidating the molecular interactions between plants and insect herbivores will deepen understanding of their interactions and also provide both a theoretical and a technical basis for new ways of managing insect pests.

Rice (*Oryza sativa*) is a crucial food crop and the primary food source for more than 50% of the world’s population. However, yield is severely threatened by various insect pests; there are three main groups of these insect pests: rice planthoppers – the brown planthopper (BPH, *Nilaparvata lugens*), white-backed planthopper (WBPH, *Sogatella furcifera*), and small brown planthopper (SBPH, *Laodelphax striatellus*); rice borers – the striped stem borer (SSB, *Chilo suppressalis*) and yellow stem borer (*Scircophaga incertulas*); and the rice leaf folder (LF, *Cnaphalocrocis medinalis*) [8, 9]. Thus far, the molecular interactions of rice with insect herbivores, especially BPH and SSB, have been extensively studied; elicitors and effectors derived from herbivores have been identified, as have signaling networks related to rice defenses and their roles, and rice defensive compounds and the mechanisms underlying their biosynthesis. In this paper, we will summarize the latest literature and identify future research directions, especially those that promote environmentally friendly pest control techniques.

## The defenses of rice in response to insect-herbivore infestation

**Fig. 1:**
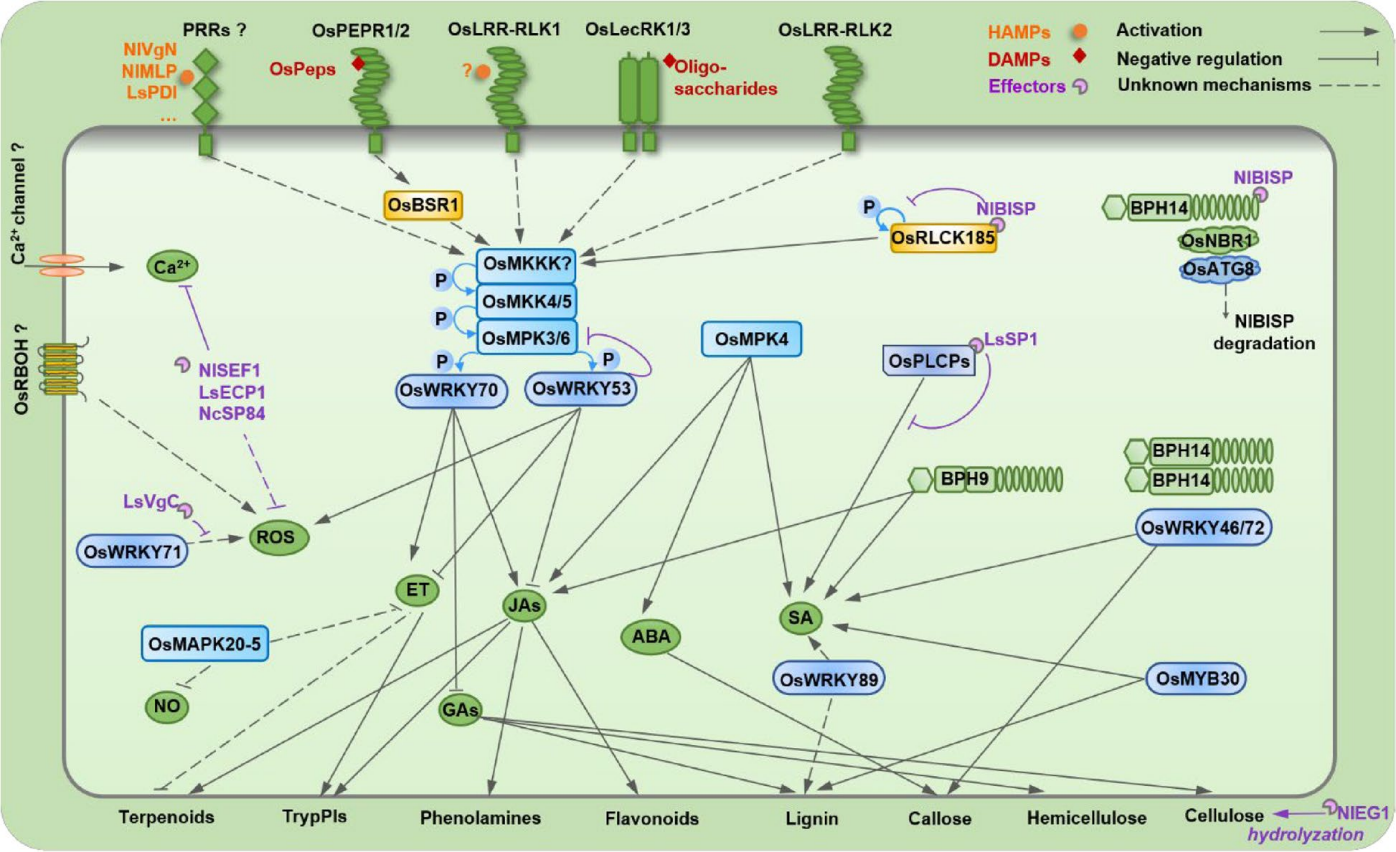
Current model of the molecular interactions between rice and herbivores. When infested by herbivores, rice plants rapidly and specifically employ pattern recognition receptors (PRRs) to perceive herbivore/damage-associated molecular patterns (HAMPs/DAMPs), initiating early signaling events. These early signaling events include the influx of calcium ions (Ca^2+^), the burst of reactive oxygen species (ROS), and the activation of mitogen-activated protein kinase (MPK) cascades. Additionally, they trigger the activation of transcription factors such as WRKYs and signaling pathways mediated by phytohormones, including jasmonic acid (JA), salicylic acid (SA), abscisic acid (ABA), ethylene (ET), gibberellins (GAs), and nitric oxide (NO). Subsequently, extensive reprogramming of the plant transcriptome, proteome, and metabolome occurs, leading to both direct and indirect resistance to rice insect pests. To cope with plant defense, adapted herbivores also secrete a repertoire of effectors or factors to suppress plant defenses or enhance the susceptibility of rice plants. However, some plants with specific resistance genes may be able to activate effector-triggered immunity by recognizing effectors, which in turn improves the ability of rice to resist herbivores. Details of the known immune receptors, HAMPs, DAMPs, effectors and signaling components are provided in the text. LRR-RLK, leucine-rich repeat receptor-like kinase; LecRK, lectin receptor-like kinase; PEPR, plant elicitor peptide (Pep) receptor; RLCK, receptor-like cytoplasmic kinase; BSR1, broad-spectrum resistance 1; RBOH, respiratory burst oxidase homologous protein; MKK, MPK kinase; MKKK, MKK kinase; PLCP, papain-like cysteine protease; NBR1, a selective autophagy cargo receptor; ATG8, autophagy associated gene 8; TrypPI, trypsin proteinase inhibitor; VgN,N-terminal subunit of vitellogenin; MLP, mucin-like protein; PDI, disulfide isomerase; BISP, BROWN PLANTHOPPER 14-interacting salivary protein; VgC, C-terminal subunit of vitellogenin; EG1, endo-β-1,4-glucanase; SEF1, salivary EF-hand calcium-binding protein1; ECP1, EF-hand calcium-binding protein 1; SP84, salivary protein 84; *Os*, *Oryza sativa*; *Nl*, *Nilaparvata lugens*; *Ls*, *Laodelphax striatellus*; *Nc*, *Nephotettix cincticeps*

When infested by herbivores, rice plants, like many other plant species, rapidly employ specific immune receptors—pattern recognition receptors (PRRs)—to perceive HAMPs/DAMPs and then initiate early signaling events [9, 10]. These events include the influx of calcium ions (Ca^2+^), a burst of reactive oxygen species (ROS), and the activation of mitogen-activated protein kinase (MPK) cascades [10–13]. Early events trigger the activation of transcription factors such as WRKYs and signaling pathways mediated by phytohormones, primarily jasmonic acid (JA), salicylic acid (SA), abscisic acid (ABA), and ethylene (ET) [14–19]. Subsequently, extensive reprogramming of the plant transcriptome, proteome, and metabolome occurs; both direct and indirect resistance of rice to herbivores results from this reprogramming [20–26] (Fig. 1).

### HAMPs and DAMPs

The initiation of an effective plant defense response relies on the plant’s ability to accurately recognize signals related to herbivores [7, 27]. These signals include DAMPs and HAMPs. DAMPs are compounds derived from plants when they are mechanically damaged or infested by insect herbivores; these compounds may be plant cell wall fragments, phytocytokines or small secondary metabolite derivatives [7, 27, 28]. HAMPs, also referred to as elicitors, are compounds derived from insect herbivores that enter plant tissues and induce defenses in plants [1, 6, 7]. Unlike elicitors, effectors are compounds secreted by insect herbivores to suppress plant defenses [2]. However, some plants with specific resistance genes may be able to activate effector-triggered immunity by recognizing effectors in turn, leading to plant resistance to herbivores [2, 29]. Therefore, effectors can also be considered as elicitors [2].

Thus far, scientists have identified various HAMPs from oral secretions (saliva and regurgitant fluids), excreta (frass or honeydew), eggs, and oviposition-associated secretions of insect herbivores, such as fatty acid‒amino acid conjugates (FACs) [30], caeliferins [31], inceptins [32], bruchins [33], benzyl cyanide [34], phosphatidylcholines [35] and an annexin-like protein [36]. These elicitors, which are mostly herbivore-specific, tend to induce specific defense responses in plants [37]. In rice herbivores, all identified HAMPs are from rice planthoppers (Table 1). By activating defense-related signaling pathways and inducing the accumulation of defensive compounds in plants, these HAMPs enhance plant herbivore resistance. The four salivary proteins of planthoppers—NlG14 [38], a mucin-like protein (NlMLP) [39], a DNAJ protein (NlDNAJB9) [40], and a disulfide isomerase (LsPDI1) [41], for example—may lead to any of the following: the activation of calcium signaling; MPK cascades and signaling pathways mediated by ROS and JA signaling; the deposition of callose; and the death of cells in the leaves of tobacco plants (*Nicotiana benthamiana*) when plants express one of these elicitor genes. Additionally, four phospholipids released by gravid WBPH females can induce the production of the ovicidal compound benzyl benzoate in rice plants; this compound eliminates WBPH eggs [42]. A recent study found that the N-terminal subunit of vitellogenins (VgN) of rice planthoppers, which is present in saliva and on the egg surface, is able to reach rice tissues, where it triggers Ca^2+^ influx and enhances the levels of hydrogen peroxide (H_2_O_2_), JA, and JA-isoleucine (JA-Ile) when the insects are feeding or during oviposition; this ability suggests that VgN reliably elicits rice defenses [17].

**Table 1:** HAMPs and effectors identified from rice herbivores and DAMPs identified from rice plants

**Name**	**Origin**	**Characterization**	**Function**	**References**
**HAMPs**				
NlMLP	*Nilaparvata lugens* salivary gland	Mucin-like protein	Induce cell death, expression of defense-related genes, and callose deposition in *Nicotiana benthamiana*	[39]
NlG14	*N. lugens* salivary gland	Uncharacterized protein	Trigger ROS accumulation, cell death, callose deposition, and activation of JA pathway in *N. benthamiana*	[38]
NlDNAJB9	*N. lugens* salivary gland	DNAJ protein	Induce the expression of defense genes, ROS accumulation, and callose deposition in *N. benthamiana*	[40]
NlVgN	*N. lugens* eggs and saliva	N-terminal subunit of vitellogenin	Induce Ca^2+^ influx, and the accumulation of H_2_O_2_, JA, and JA-Ile in rice plants	[18]
Microbes	*N. lugens* honeydews	Honeydew-associated microbes	Induce accumulation of phytoalexins in rice leaves and release of volatile organic compounds from rice	[43]
LsPDI1	*Laodelphax striatellus* salivary gland	Disulfide isomerase	Induce ROS bursts, cell death, callose deposition, and activation of JA pathway	[41]
Phospholipids	*Sogatella furcifera* females	Phospholipids	Promote the production of benzyl benzoate in rice plants to eliminate eggs of *S. furcifera*	[42]
*N*-linolenoyl-L-Gln (Gln-18:3)	*Mythimna loreyi* oral secretions	Fatty acid–amino acid conjugate	Induce the production of ROS and phytoalexins (*p*-coumaroylputrescine and momilactone B)	[44]
Unknown	*M. loreyi* oral secretions	Unknown protein (> 3.5 kDa)	Induce the production of ROS and phytoalexins (*p*-coumaroylputrescine and momilactone B)	[44]
**DAMPs**				
Oligosaccharides	*Oryza sativa*	Hydrolysates of the Poaceae-specific metabolite mixed-β-1,3;1,4-D-glucans	Induce JA-mediated rice defenses	[45]
OsPep3	*O. sativa*	C-termini of PRECURSORs of PEP (PROPEP) proteins	Induce activation of MPK cascade, and the production of ROS, JA-Ile, and defensive compounds	[46]
**Effectors**				
Kat-1	*N. lugens* salivary gland	Catalase	Decompose H_2_O_2_	[47]
NlEG1	*N. lugens* salivary gland	Endo-β-1,4-glucanase	Degrade celluloses in rice	[48]
NlSEF1	*N. lugens* salivary gland	EF-hand calcium-binding protein	Suppress the Ca^2+^ influx and H_2_O_2_ production	[16]
Nl12	*N. lugens* salivary gland	Disulfide isomerase	Induce cell death in *N. benthamiana*	[49]
Nl16	*N. lugens* salivary gland	Apolipophorin-III protein	Induce cell death in *N. benthamiana*	[49]
Nl28	*N. lugens* salivary gland	Cysteine-rich protein	Induce cell death in *N. benthamiana*	[49]
Nl32	*N. lugens* salivary gland	Chemosensory protein	Induce a dwarf phenotype of *N. benthamiana* plants	[49]
Nl40	*N. lugens* salivary gland	*N. lugens*-specific salivary protein	Induce chlorosis in *N. benthamiana*	[49]
Nl43	*N. lugens* salivary gland	Uncharacterized protein	Induce cell death in *N. benthamiana*	[49]
NlSP7	*N. lugens* salivary gland	*N. lugens*-specific salivary protein	Mediate tricin metabolism in rice	[50]
NlugOBP11	*N. lugens* salivary gland	odorant-binding protein	Inhibit SA levels in rice protoplasts or *N. benthamiana* leaves	[51]
NlHSC70-3	*N. lugens* salivary gland	heat shock 70 kDa protein cognate 3	Suppress bacterial elicitor flg22-induced ROS bursts and expression of defense-related genes in *N. benthamiana*	[52]
LsECP1	*L. striatellus* salivary gland	EF-hand calcium-binding protein	Suppress the Ca^2+^ influx and H_2_O_2_ production in rice protoplasts or *N. benthamiana* leaves	[53]
DNase II	*L. striatellus* salivary gland	Deoxyribonuclease II	Suppress H_2_O_2_ and callose accumulation in rice	[54]
LsVgC	*L. striatellus* salivary gland	C-terminal subunit of vitellogenin	Suppress H_2_O_2_ accumulation by targeting OsWRKY71 in rice	[55]
LsSP1	*L. striatellus* salivary gland	Salivary glands-specific protein	Reduce SA-mediated rice defenses by interacting with papain-like cysteine proteases in rice	[56]
NcSP84	*Nephotettix cincticeps* salivary gland	EF-hand calcium-binding protein	Bind Ca^2+^ and prevent Ca^2+^-mediated rice defenses	[57]
Unknown	*Chilo suppressalis* oral secretions	Microbes isolated from *C. suppressalis* oral secretions	Suppress JA-mediated rice defenses	[58]

Interestingly, some microbes in BPH honeydews also induce the accumulation of phytoalexins and the release of volatiles from plants, thereby enhancing the resistance of rice to the insect [43]. These results imply that, in addition to perceiving HAMPs directly from insect herbivores, plants can also perceive these compounds from herbivore-associated microbes and, in response, initiate defenses. Other rice insect herbivores, such as SSB, *Parnara guttata*, *Mythimna loreyi*, *Spodoptera frugiperda*, *Spodoptera mauritia*, and *Nephotettix bipunctatus*, have also been reported to activate strong defense responses in rice [44, 59–62]. However, no HAMPs from these insect herbivores have been identified.

In addition to HAMPs, DAMPs also play an important role in regulating plant defenses [28, 63]. Thus far, many DAMPs have been identified from host plants, and their roles in mediating plant-herbivore interactions have also been extensively studied, especially those of phytocytokines, systemin [64] and plant elicitor peptides (Peps) [65]. Systemin, an 18-amino acid peptide that is cleaved from precursor prosystemins, specifically activates local and systemic defense responses in plants of the Solanaceae by regulating the JA-signaling pathway and the biosynthesis of trypsin proteinase inhibitors (TrypPIs) [28]. Peps, cleaved from the precursor PROPEPs, were initially thought to be defense signals that amplify immunity in Arabidopsis. Further studies found that peps are also present in Gramineous, Solanaceous, and Fabaceous plants, such as rice, *Zea mays*, *Solanum lycopersicum*, and *Glycine max* [45, 65–67]. In rice, two DAMPs, OsPep3 [45, 67] and a class of oligosaccharides [46], have been identified (Table 1). Both treatment with *M. loreyi* oral secretions and BPH infestation significantly induced the expression of the OsPep3 precursor gene *OsPROPEP3* in rice; moreover, the exogenous application of OsPep3 amplified herbivore-induced rice defense responses, including the activation of OsMPK3/6 and the production of ROS, JA-Ile, and defensive compounds (*p*-coumaroylputrescine, feruloyl putrescine, momilactone A and momilactone B). Overall, the exogenous application of OsPep3 increases plant resistance to both insect pests and pathogens [45, 67]. Recently, oligosaccharides—the hydrolysates of the Poaceae-specific secondary metabolite mixed-β-1,3;1,4-D-glucans (MLGs)—have been found to contribute to the ability of rice to resist BPH; the infestation of rice by this species not only promotes the hydrolysis of MLGs into oligosaccharides but also induces the binding of OsMYC2, a key transcription factor of JA signaling, to the promoter of the MLG biosynthesis-related gene *OsCsIF6*, thereby increasing the production of MLGs and extending rice defense responses [46].

### Plant immune receptors

Plants use different immune receptors to monitor infection/infestation signals in both extracellular and intracellular compartments. These immune receptors include plasma membrane-localized PRRs, which mainly perceive extracellular signals, such as HAMPs, and intracellular resistance (R) proteins, which mainly perceive intracellular signals, such as effectors [2, 29, 68, 69] (Fig. 1).

#### Plasma membrane-localized PRRs

Plasma membrane-localized PRRs are categorized into receptor-like kinases (RLKs) or receptor-like proteins (RLPs) [2, 28, 29, 68]. RLKs typically comprise a ligand-binding extracellular domain, a transmembrane domain, and a cytoplasmic kinase domain, whereas RLPs lack a cytoplasmic kinase domain [29, 68]. After PRRs perceive a ligand, they associate with co-receptors, such as somatic embryogenesis receptor kinases (SERKs), and activate downstream signaling events [7, 27, 29, 68]. To date, only one leucine-rich repeat receptor-like protein (LRR-RLP) in plants has been identified as a PRR that perceives HAMPs. This PRR, known as inceptin receptor (INR), is present in plants of the common bean, *Vigna unguiculata*, and related legumes, and recognizes inceptin, a HAMP from the oral secretions of Lepidopteran caterpillars [70]. INR constitutively interacts with coreceptors, such as the brassinosteroid insensitive 1-associated kinase 1 (BAK1) (one of the SERKs), and binds to the adaptor RLK, suppressor of BAK1-interacting receptor-like kinase 1 (SOBIR1); subsequently, the INR-containing complex activates the following defense responses, such as enhancing the levels of ROS, ET, peroxidases, and TrypPIs, and then confers the ability to resist insect herbivores on plants [70]. In rice, a leucine-rich repeat receptor-like kinase (OsLRR-RLK1) has been identified as a putative plant PRR that might perceive HAMPs from SSB larvae. Remarkably, silencing *OsLRR-RLK1* has been shown to impair the level of SSB-induced OsMPK3/6 phosphorylation and decrease SSB-elicited, but not wounding-elicited, levels of JA, ET, and TrypPI activity and the resistance of rice to SSB [10]. Notably, using multiple forward genetics approaches, an LRR-RLK ZmFACS (a homolog of OsLRR-RLK1) in *Z. mays* was identified as a candidate PRR responding to Gln-18:3, a typical FAC isolated from Lepidoptera larval oral secretions [71]. Considering that ZmFACS-mediated plant antiherbivore defenses are similar to the defenses mediated by OsLRR-RLK1 in rice, it is possible that both ZmFACS and OsLRR-RLK1 function as PRRs, sensing specific FACs. The association of direct receptors with FACs has not yet been demonstrated.

Unlike the study of HAMP-recognizing PRRs, which is quite new, the study of DAMP-recognizing PRRs has made great progress. Thus far, several DAMP-recognizing PRRs, such as those that recognize systemin [72] and those that recognize Peps [73–75], and their underlying mechanisms have been reported. AtPEPR1 in *Arabidopsis thaliana*, for instance, interacts with AtPep1 ~ 6 and mainly controls the activation of foliar AtPep signaling, whereas AtPEPR2 binds AtPep1/2 and modulates AtPep signaling in plant roots [73–75]. Although infestation by *S. littoralis* larvae strongly enhances the transcript levels of *AtPEPR1*, *AtPEPR2*, and *AtPEOPEP3* in Arabidopsis, *pepr1 pepr2* double mutant plants are highly insensitive to Peps and display a reduced defense response and less resistance to *S. littoralis* [75]. In rice, the overexpression of *OsPEPR1* further enhances rice defenses against *M. loreyi* induced by wounding and treatment with OsPep3 [67]. In contrast, OsPep3-elicited rice defenses against BPH are abolished when *OsPEPR1/2* is knocked out [45]. This research suggested that the Pep-PEPR ligand-receptor signal module is conserved across different plant species and imparts plant resistance when DAMPs are sensed. Additionally, *Bph3*, a gene cluster comprising three genes encoding lectin receptor-like kinases (OsLecRK1/2/3), was identified as the first potential RLK gene contributing to rice defense against BPH [76]. A recent study revealed that MLG-derived oligosaccharides directly bind to OsLecRK1 and OsLecRK3, thereby enhancing OsLecRK activity [46]; in contrast, oligosaccharide-induced rice resistance to BPH in the MLG biosynthesis gene (*OsCsIF6*)-overexpressing rice plants is nullified when *OsLecRK1/3* is knocked out [46]. This finding suggested that OsLecRK1/3 functions similarly to PRRs in rice plants: it perceives MLG-derived oligosaccharides and initiates oligosaccharides-triggered rice defenses.

#### Intracellular R proteins

When plants defend against HAMPs, adapted insect herbivores can employ effectors to overcome these defenses (see section ‘Regulation of rice defenses and susceptibility by insect herbivores’). Furthermore, some plants develop intracellular immune receptors, also known as R proteins, to detect these effectors and thus initiate effector-triggered defenses [2, 29, 68, 69]. The largest group of intracellular R proteins belong to the nucleotide-binding site leucine-rich repeat proteins (NB-LRRs, also termed NLRs) [2, 69]. NLRs contain three distinct domains: a nucleotide-binding (NB-ARC) domain, a C-terminal leucine-rich repeat (LRR) domain, and an N-terminal extension domain [69]. Several *R* genes encoding intracellular NLRs conferring plant resistance to root-knot nematodes, such as *PcMa* from *Prunus cerasifera* [77], *PsoRPM2* from *Prunus sogdiana* [78], *AtDSC1* from Arabidopsis [79], and *SlMi* from *Solanum lycopersicum* [80], have been cloned.

In rice, two intracellular R proteins that confer the ability to resist BPH infection in rice have been cloned and characterized; these R genes include *Bph14* and *Bph9* (with alleles *Bph1*, *Bph2*, *Bph7*, *Bph10*, *Bph18*, *Bph21*, and *Bph26*) [81–86]. Among these R proteins, BPH14 activates ROS and SA-mediated signaling pathways, leading to an increase in the production of TrypPIs; moreover, the coiled-coil (CC) and NB-ARC domains of BPH14 interact with and stabilize OsWRKY46 and OsWRKY72, promoting the expression of callose synthase genes, enhancing callose deposition, and eventually reinforcing rice resistance [81, 84]. Significantly, a recent study reported a vital role of BPH14 in sensing BPH effectors and mediating trade-offs between rice growth and defense [85]. BPH secretes the effector BPH14-interacting salivary protein (BISP) into rice plants during feeding. In susceptible plants, BISP targets a receptor-like cytoplasmic kinase (RLCK), OsRLCK185, and inhibits the autophosphorylation of OsRLCK185, thereby decreasing basal defenses in rice. In *Bph14*-carrying resistant plants, BPH14 directly binds to BISP, activating effector-triggered rice defenses but inhibiting rice growth. Notably, the BISP-BPH14 module also binds to the selective autophagic cargo receptor OsNBR1. As insects cease feeding and depart from these sites, the activated autophagy pathway rapidly degrades BISP, thereby maintaining an appropriate level of BISP-BPH14 module-triggered rice defenses and restoring rice growth [85]. This study elucidates the molecular mechanism underlying the trade-offs in rice between defense and growth in the BISP-BPH14-OsNBR1 module. Additionally, BPH9 confers resistance to BPH in rice by modulating the SA-, JA-, and ET-signaling pathways [82]. However, which effectors are perceived by BPH9 cells remains unknown.

### Early signaling events

Following ligand perception, plant immune receptors activate multiple kinases, mainly including RLCKs; activated RLCKs phosphorylate downstream factors, such as Ca^2+^ channel proteins, respiratory burst oxidase homologous (RBOH) proteins and MPK kinase kinases (MKKKs), thereby activating a series of follow-up responses, such as Ca^2+^ influx, an ROS bursts, and MPK cascades [2, 7, 29] (Fig. 1). These early signaling events play critical roles in plant defense responses to different stimuli, including herbivore infestation [87–89].

In rice, herbivore infestation also activates various early signaling events (Fig. 1). Rice planthopper infestation, for instance, induces bursts of cytosolic Ca^2+^ and ROS (predominantly H_2_O_2_) and activates MPK cascades [13, 17, 81]. SSB infestation also quickly and strongly activates MPKs [10, 90]. Moreover, RLCKs also play an important role in regulating these early signaling events in rice. After chitin or peptidoglycan (PGN) treatment, rice chitin and PGN receptors, chitin oligosaccharide elicitor-binding protein (OsCEBiP), and lysin motif (LysM)-containing proteins (OsLYP4 and OsLYP6) form a complex by interacting with the co-receptor chitin elicitor receptor kinase 1 (OsCERK1). Then, the complex triggers the activation of OsRLCK185 and OsRLCK176 [91]. Subsequently, activated OsRLCK185 not only directly phosphorylates the cyclic nucleotide gated channel (OsCNGC9), facilitating channel opening and inducing Ca^2+^ influx but also activates the OsMKKK18/24-OsMKK4-OsMPK3/6 cascade [91, 92]. Moreover, OsRLCK185 regulates the activation of RBOHs, leading to the ROS bursts [91, 92]. Like OsRLCK185, OsRLCK176 also positively regulates chitin/PGN-induced ROS bursts, Ca^2+^ influxes, and OsMPK3/6 activities [93]. Another RLCK, *BSR1*, positively regulates *M. loreyi*- or OsPep3-elicited rice defenses, including the expression of defense genes, and the production of H_2_O_2_ and diterpenoid phytoalexins, thus enhancing the ability of rice to resist feeding on *M. loreyi* larvae [94].

MPK cascades, one of the most highly conserved signaling modules downstream of PRR complexes, have also been reported to play an important role in regulating defense-related phytohormone signaling pathways [95, 96]. In rice, several MPK members have been revealed to participate in herbivore-induced defenses (Fig. 1). For instance, OsMPK3 and OsMPK4 contribute to rice defenses against SSB by positively regulating the JA-, SA-, and ET-signaling pathways and the production of TrypPIs [90, 97]. The OsMEK4-OsMPK3/6 module functions downstream of *OsLRR-RLK1* and *OsLRR-RLK2*; silencing *OsLRR-RLK1* or *OsLRR-RLK2* significantly decreases the basal and herbivore-induced activation of OsMPK3/6 and induces JA and ET in rice, making rice susceptibility to SSB larvae [10, 98]. In addition to their role in regulating the ability of rice to resist SSB, some MPKs also play a role in rice defenses against rice planthoppers. The overexpression of *OsMKK3* enhances BPH-induced JA, JA-Ile, and ABA levels and improves the resistance of rice to BPH [99]. The infection of gravid BPH females quickly induces significant levels of *OsMAPK20-5* transcripts, but infestation by BPH nymphs has little effect; silencing *OsMAPK20-5* enhances BPH oviposition but not feeding-induced nitric oxide (NO) and ET accumulation, which in turn reduces the hatching rate of BPH eggs and rice tolerance to infestation by gravid BPH females [100]. These results indicate that *OsMAPK20-5* protects plants from autotoxicity by suppressing herbivore-induced defense signaling pathways, which are mainly mediated by NO and ET.

### Phytohormone-mediated signaling pathways

Phytohormone-mediated signaling pathways function downstream of early signaling events and control plant defense outputs, including the reconfiguration of the transcriptome and metabolome, and plant resistance to insect herbivores. In rice, herbivore infestation can induce changes in the profile of phytohormones, such as JAs, SA, ET, and ABA, all of which jointly regulate the ability of rice to resist insect herbivores [10, 18, 24, 60, 101] (Fig. 1).

As reported in many plant species, the JA-signaling pathway also plays an important role in regulating the resistance of rice to insect herbivores. In general, the JA-signaling pathway positively regulates the resistance of rice to chewing insects, such as SSB and LF [12, 102–108], whereas its role in the resistance of rice to piercing-sucking insects, such as rice planthoppers, varies. For instance, overexpression of genes related to JA biosynthesis, such as an allene oxide cyclase gene (*OsAOC*) or a *cis*-12-oxo-phytodienoic acid (OPDA) reductase 3 (*OsOPR3*), in rice enhances the resistance of plants to SSB larvae [102], whereas silencing or knocking out genes related to JA biosynthesis, such as a lipoxygenase gene (*OsHI-LOX*) [103], phospholipase D genes (*OsPLDα4* and *OsPLDα5*) [104], and allene oxide synthase genes (*OsAOS1* and *OsAOS2*) [12], decreases resistance to SSB. The knockout of *OsAOC* in rice decreases the levels of herbivore-induced defensive compounds, including some phenolamines, flavonoids, and herbivore-induced plant volatiles (HIPVs); lower levels of these compounds increase the growth of rice LF larvae [105]. Additionally, the JA receptor coronatine insensitive 1a (OsCOI1a) [106] and the core transcription factor of the JA-signaling pathway (OsMYC2) [105] have been shown to positively regulate rice defenses against rice LF larvae. Both positive and negative regulation of the JA-signaling pathway in rice planthopper resistance have been reported. Knocking out *OsAOC* [24], JA receptors (*OsCOI1a* and *OsCOI2*) [106, 109] or the *OsMYC2* transcription factor of the JA-signaling pathway [24] in rice, for instance, decreases the hatching rate of BPH eggs; moreover, silencing *OsMYC3* (the homolog of *OsMYC2*) in rice facilitates the feeding of WBPH and SBPH nymphs [110]. However, silencing genes related to JA biosynthesis, such as *OsAOS1*, *OsAOS2*, or *OsHI-LOX*, in rice enhances the ability of rice to resist rice planthoppers [12, 103]. This discrepancy might be related to the genetic background of the rice varieties used and to the genes that were knocked out or silenced. Many genes have multiple functions; therefore, silencing such a gene may also influence plant insect resistance by affecting other pathways.

The ET-signaling pathway also contributes to the ability of rice to resist insect herbivores; however, its role changes with herbivore species [19, 111–113]. Silencing *OsACS2*, a gene encoding a key enzyme related to ET biosynthesis, ACC (1-amino-1-cyclopropanecarboxylic acid) synthase, decreases the production of SSB-elicited ET, TrypPIs, and HIPVs, and decreases the resistance of rice to SSB larvae [19]. However, silencing *OsACS2* enhances the production of two BPH-induced rice volatiles, 2-heptanone and 2-heptanol; higher levels of these volatiles subsequently repel the feeding and oviposition of gravid BPH females but enhance the attractiveness of the rice plant to the egg parasitoid of BPH [19]. The negative effect of the ET pathway on the regulation of rice BPH resistance has also been reported in other studies. Ethylene-insensitive3-like1 (OsEIL1) [112] and OsEIL2 [111], two key ET-responsive transcription activators, have been reported to negatively affect the ability of rice to resist BPH; moreover, the E3 ligases, OsEBF1 (ethylene-insensitive3-binding F-box protein 1) and OsEBF2, positively modulate the ability of rice to resist BPH by directly interacting with OsEIL1 and degrading it through the ubiquitination pathway [112, 113]. Recently, the role of the ET pathway in regulating the light-induced ability of rice to resist BPH has been reported [111]: light facilitates the accumulation of the phytochrome protein (OsPhyB) in rice nuclei and accelerates the degradation of the phytochrome-interacting factor protein (OsPIL14); both processes decrease ET production and OsEIL2 protein levels, ultimately enhancing the resistance of rice to BPH.

The SA-signaling pathway can regulate the direct and indirect defenses of rice against planthoppers and LFs. For example, the exogenous application of methyl salicylate (MeSA) to rice plants not only restricts the growth, development, and reproduction of rice LFs [114], but also attracts the natural enemies of rice planthoppers [11, 115]. In BPH-resistant rice varieties, feeding by BPH nymphs significantly induces the expression of genes encoding rate-limiting enzymes involved in SA biosynthesis, like isochorismate synthase (*OsICS1*) and L-phenylalanine ammonia-lyase genes (*OsPALs*) [116]. The suppression of *OsICS1* in rice enhances the feeding preference of BPH females for plants [117]; moreover, silencing *OsPALs* reduces BPH feeding-induced SA and lignin levels, compromising the ability of rice to tolerate BPH nymphs [116]. Rice plants carrying the BPH resistance gene *Bph14* exhibit high SA levels, which leads to increased levels of BPH nymph-induced callose in plants, thereby enhancing rice resistance [84, 85].

In addition to signaling pathways mediated by JA, ET, and SA, pathways mediated by ABA, gibberellins (GAs) and cytokinins (CKs) also play roles in rice defense against insect herbivores [118–123] (Fig. 1). ABA levels are increased in rice plants by exogenously applying ABA, overexpressing the ABA biosynthesis-related gene *OsNCED3* (9-*cis*-epoxycarotenoid dioxygenase), or silencing the ABA catabolic gene *OsABA8ox3* (ABA 8’-hydroxylase), for instance, to effectively promote callose deposition in plant sieve plates and prevent BPHs from ingesting phloem sap [118, 119]. Similarly, strengthening the GA pathway in rice, by, for example, exogenously applying GA_3_ [120], expressing the GA receptor gene *OsGID1* [121], or silencing the GA signaling suppressor DELLA gene *OsSLR1* [122], enhances lignin and cellulose content, which in turn fortifies cell walls and ultimately enhances the ability of rice to resist gravid BPH females. BPH feeding also significantly induces the accumulation of *cis*-zeatin-type and *N*^6^-(Δ^2^-isopentenyl) adenine-type CKs in rice [123]. Treating rice with 6-benzylaminopurine (6-BA, a synthetic CK analog) or knocking out *OsCKX1* (a CK oxidase/dehydrogenase gene that inactivates CKs) in rice facilitates the accumulation of CKs, JAs (JA-Ile and JA-Val), and lignin, leading to an increase in rice tolerance to BPH [123].

Accumulated evidence suggests that crosstalk plays an important role in regulating herbivore-induced rice defenses. For example, although some studies have found that JA signaling positively regulates the ability of rice to directly and indirectly resist planthoppers, as described above, other research has also revealed that silencing genes related to JA biosynthesis, such as *OsAOS1*, *OsAOS2*, or *OsHI-LOX* in rice, enhances the ability of rice to resist rice planthoppers by increasing the levels of SA and ROS [12, 103]. Silencing the SA-receptor gene *OsNPR1* increases the resistance of rice to SSB by activating JA- and ET-mediated signaling pathways [124]. Additionally, the ET-responsive transcription activator OsEIL1 directly binds to the promoter of *OsLOX9* (*OsHI-LOX*) and negatively regulates rice BPH resistance [112]. When JA biosynthesis is blocked in rice, 6-BA-induced rice tolerance to BPH is abolished, suggesting that the ability of CK-mediated rice to resist BPH depends on the JA pathway [123].

### Transcription factors

Plant defense responses are accompanied by the activation and suppression of defense genes, with transcription factors (TFs) playing a crucial role in this process [4, 6, 7]. Hitherto, TFs associated with rice defense responses have been reported to be mainly in the WRKY, AP2/ERF (APETALA2/ethylene responsive factor), MYB, bZIP (basic leucine zipper factor), and bHLH (basic helix-loop-helix) subfamilies (Fig. 1).

WRKY TFs are essential components that function as both up- and downstream phytohormone signals in herbivore-induced rice defenses. Both OsWRKY53 and OsWRKY70, for example, interact directly with and are phosphorylated by OsMPK3/6 [14, 15], but their roles in mediating defensive responses in rice differ: OsWRKY70 positively regulates the SSB-induced accumulation of JA, ET, and TrypPIs, and the resistance of rice to SSB [15], whereas OsWRKY53 inhibits OsMPK3/6 activity and subsequent JA/ET-mediated rice defense responses, thereby maintaining an appropriate level of SSB-induced rice defenses [14]. Moreover, OsWRKY53 enhances the resistance of rice to BPH by increasing H_2_O_2_ production and reducing ET accumulation [14], but OsWRKY70 negatively modulates GA biosynthesis and the ability of rice to resist BPH [15]. OsWRKY46/72 directly binds to the promoter of the callose-biosynthesis gene *LOC_Os01g67364.1*, thereby enhancing callose deposition [84]. *OsWRKY89* overexpression positively contributes to the resistance of rice to WBPH by regulating the lignin content in rice [125]. Silencing the SA-responsive gene *OsWRKY45* confers on rice with the ability to resist BPH in both greenhouses and in the field by enhancing H_2_O_2_ levels in rice [126].

In addition to WRKYs, other TFs also play roles in regulating the ability of rice to resist insect herbivores. OsMYB22, for instance, is a transcriptional repressor that interacts with the TOPLESS transcriptional co-repressors and recruits a histone deacetylase (OsHDAC1); the result is a tripartite complex that suppresses the expression of the *F3’H* (flavanone 3’-hydroxylase) gene (a negative modulator of rice resistance to BPH), thereby enhancing the ability of rice to tolerate BPH [127]. OsMYB30 activates the transcription of *OsPAL6/8* by directly binding to the promoters of these two genes, and contributes to the production of SA and lignin, as well as to the ability of rice to tolerate BPH nymphs [116]. *OsERF3* (ethylene-responsive factor 3) and *OsDREB1A* (dehydration-responsive element-binding gene 1A) belong to the AP2/ERF subfamily [128, 129]. Silencing *OsERF3* decreases the expression of *OsMKK3*, *OsMPK3*, and *OsWRKY53/70* in rice, thereby inhibiting the JA-mediated defense responses of rice to SSB and enhancing the susceptibility of rice to SSB larvae [128]. The suppression of *OsDREB1A* enhances BPH-induced JA, JA-Ile, and ABA levels, and decreases ET production in rice, which subsequently decreases hatchability and delays the developmental duration of BPH eggs [129]. OsMYC2, a member of the bHLH- subfamily TF, activates the expression of *OsNOMT* (naringenin 7-*O*-methyltransferase, which catalyzes naringenin to yield sakuranetin) and *OsLIS* (linalool synthase), as well as the production of sakuranetin and linalool [130]. Moreover, the transactivation activity of OsMYC2 could be enhanced by other TFs, such as OsMYC2-like protein 1 (OsMYL1, also termed OsMYC3), OsMYL2 (also termed OsMYC4) and OsRERJ1, by physical interactions [130, 131].

### Defensive compounds

Herbivore-induced changes in phytohormone profiles usually lead to an increase in defensive compounds and a decrease in nutrients in plants, which in turn enhances the direct and indirect resistance of plants to insect herbivores [3, 7, 26]. Plant defensive compounds mainly include terpenoids, phenolics, alkaloids, phenolamines, defensive proteins, HIPVs, and organic acids and their salts [7, 132]. These are thought to defend against insect herbivores by limiting their nutrient access, poisoning them, impairing their ability to digest food, and attracting their natural enemies, among other strategies [3, 26, 133–135].

In rice, some defensive compounds against insect herbivores have been reported (Table 2). They can influence the behavior, feeding, growth, development, survival, or fecundity of insect herbivores. Some organic acids and their salts (aconitic acid, oxalic acid, calcium oxalate, and sodium oxalate, among others), for instance, have been found to restrict the feeding of rice planthoppers [136, 137]. Additionally, certain flavonoids (for example, tricin [138], sakuranetin [139], quercitrin [140], schaftoside [141], isoschaftoside, neoschaftoside [142], and epigallocatechin [21]), a phenolic acid (ferulic acid) [143], and some phenolamines (feruloyl putrescine, isopentylamine,* p*-coumaroyl putrescine, *N*-feruloyl tyramine, feruloyl agmatine, and *N1*,*N10*-diferuloyl spermidine [61, 144]) significantly influence the survival and development of rice planthoppers. The actions of different defensive compounds exhibit different mechanisms. Schaftoside, for example, robustly binds with cyclin-dependent kinase 1 of BPH (NlCDK1) and then inhibits the activation of NlCDK1 as a kinase [145]. Some phenolic acids, including ferulic acid, vanillic acid, and 4-hydroxybenzoic acid, inhibit the acetylcholinesterase activity in rice weevils, delaying their molting process [146]. Sakuranetin directly impedes the growth of beneficial endosymbionts in BPH and disrupts the nutritional balance essential for BPH development [139]. Lignin and polysaccharides, such as cellulose, hemicellulose, pectin, and callose, bolster the rigidity of plant cell walls, increasing the difficulty of herbivore feeding and digestion [83, 84, 119].

**Table 2:** Defensive compounds identified from rice plants

**Chemicals**	**Function**	**References**
Silicic acid	Inhibit planthopper feeding	[136]
Potassium oxalate	Inhibit planthopper feeding	[136]
Sodium oxalate	Inhibit planthopper feeding	[136]
Oxalic acid	Inhibit planthopper feeding	[137]
Maleic acid	Inhibit planthopper feeding	[137]
*Trans*-aconitonic acid	Inhibit planthopper feeding	[137]
Tricin	Decrease the feeding activity and survival rate of *Nilaparvata lugens* nymphs, and the fecundity of their females	[138]
Sakuranetin	Impede the growth of beneficial endosymbionts in *N. lugens*; disrupt the nutritional balance essential for its development; enhance the tolerance of rice plants to their nymphs	[139, 147]
Quercitrin	Decrease the survival rate of *Sogatella furciferas* nymphs; enhance the tolerance of rice plants to *S. furciferas* nymphs	[140]
Schaftoside	Bind with cyclin-dependent kinase 1 of *N. lugens* to inhibit its activation as a kinase by suppressing phosphorylation on its Thr-14 site; inhibit the ingestion activity of their females; decrease the survival and development of their females	[141, 142]
Isoschaftoside	Inhibit the feeding of *N. lugens* females; decrease their survival and development	[142]
Neoschaftoside	Inhibit the feeding of *N. lugens* females; decrease their survival and development	[142]
Epigallocatechin	Decrease the survival rate and weight gain of *N. lugens* nymphs	[21]
Sinapyl alcohol	Decrease the survival rate and weight gain of *N. lugens* nymphs	[21]
Ferulic acid	Inhibit the acetylcholinesterase activity in rice weevils, thereby delaying their molting process; decrease the survival rate of *N. lugens* nymphs	[143]
Isopentylamine	Inhibit the feeding of and decrease the survival rate of *N. lugens* adults	[148]
Feruloyl putrescine	Decrease the survival rate of *N. lugens* and *S. furciferas* females	[61, 144]
*p*-coumaroyl putrescine	Decrease the survival rate of *N. lugens* females	[61]
*N*-feruloyl tyramine	Decrease the survival rate of *S. furciferas* females	[144]
Feruloyl agmatine	Decrease the survival rate of *S. furciferas* females	[144]
*N1,N10*-diferuloyl spermidine	Decrease the survival rate of *S. furciferas* females	[144]
Lignin	Bolster the rigidity of plant cell walls; increase the difficulty of herbivore feeding and digestion	[116, 125]
Cellulose	Bolster the rigidity of plant cell walls; increase the difficulty of herbivore feeding and digestion	[122]
Hemicellulose	Bolster the rigidity of plant cell walls; increase the difficulty of herbivore feeding and digestion	[122]
Callose	Prevent rice planthoppers from ingesting phloem sap	[81, 84, 118, 119]
Polyphenol oxidase	Oxidize polyphenols to form ROS and quinones, which bind to amino acids or proteins, significantly reducing their availability to herbivores	[135]
Peroxidase	Oxidize polyphenols to form ROS and quinones, which bind to amino acids or proteins, significantly reducing their availability to herbivores; increase the lignification of cell walls, thereby increasing the difficulty of herbivore feeding and digestion	[45, 135]
Phenylalanine ammonia-lyase	Function as a key enzyme in the biosynthesis of salicylic acid and phenylpropanoid compounds in rice plants	[116, 149]
Trypsin proteinase inhibitors	Inhibit the activity of digestive enzymes in the midgut of multiple rice pests, thereby impeding insect development	[103, 128, 150]
Xylanase-inhibiting proteins	Reduce the weight gain of *Chilo suppressalis* larvae, and the feeding and oviposition preference of *N. lugens* females	[151]
Momilactone B	Decrease the weight gain of *Mythimna loreyi* larvae	[94]
Benzyl benzoate	Eliminate *S. furcifera* eggs	[42]
2-Heptanone	Repel *N. lugens* females; attract *Anagrus nilaparvatae*	[19, 150, 152]
2-Heptanol	Repel *N. lugens* females; attract *A. nilaparvatae*	[19, 150, 152]
*S*-Linalool	Repel *N. lugens* females; attract *Cnaphalocrocis medinalis*, *A. nilaparvatae*, *Cotesia chilonis* and *Cyrtorhinus lividipennis*	[153, 154]
2-Nonanone	Repel *A. nilaparvatae*	[152]
Isopropyl myristate	Repel *A. nilaparvatae*	[152]
2-Tridecanone	Repel *A. nilaparvatae*	[152]
(*E*)-Hept-2-enyl acetate	Repel *A. nilaparvatae*	[152]
α-Pinene	Repel *A. nilaparvatae*	[152]
α-Zingiberene	Repel *A. nilaparvatae*	[155]
(+)-Limonene	Attract *S. furcifera*; repel *A. nilaparvatae*	[156]
β-Farnesene	Attract *S. furcifera*, *Laodelphax striatellus* and *Telenomus podisi*	[156]
β-Myrcene	Attract *T. podisi*	[156]
(*E*)-2-Hexenal	Repel *N. lugens*; attract *C. medinalis*; enhance the oviposition preference of *S. furcifera* females and promote the development of their eggs	[152, 157]
(*Z*)-3-Hexenal	Repel *N. lugens* females and inhibit the development of their eggs; enhance the oviposition preference of *S. furcifera* females and promote the development of their eggs; attract *T. podisi*	[115, 157, 158]
(*Z*)-3-Hexen-1-ol	Repel *N. lugens* females and inhibit the development of their eggs; attract *C. medinalis*; enhance the oviposition preference of *S. furcifera* females and promote the development of their eggs	[157, 158]
(*Z*)-3-Hexenyl acetate	Attract *C. medinalis*	[115, 159]
(*E*)-β-caryophyllene	Attract *N. lugens* females and promote the development of *N. lugens* nymphs; attract *A. nilaparvatae* and *Trigonotylus caelestialium*	[152, 153, 160]
(*E,E*)-4,8-dimethyl-1,3,7-nonanetriene	Attract *A. nilaparvatae* and *C. chilonis*	[152, 154]
(*E,E,E*)-4,8,12-trimethyltrideca-1,3,7,11-tetraene	Attract *A. nilaparvatae* at low concentrations but repel at the high concentrations; attract *C. chilonis*	[152, 154]
Methyl benzoate	Repel *S. frugiperda* females and inhibit their fecundity; attract *C. marginiventris*	[159]
Methyl salicylate	Repel *N. lugens*; attract *A. nilaparvatae*; repel *C. chilonis* at low concentrations but attract at high concentrations	[115, 152, 154]

Plant defensive proteins, such as polyphenol oxidases (PPOs), peroxidases (PODs), and PALs, can modify plant cell walls and compounds, and confer the ability to resist insect herbivores on plants [135]. For example, PODs and PPOs can modify the structure of polyphenols and convert them into quinones, which bind to amino acids or proteins, reducing the availability of nutrients to insect herbivores [135]. Moreover, PALs are key enzymes related to the biosynthesis of salicylic acid and phenylpropanoids in rice plants [149]. PODs and PALs are required for the lignification of plant cell walls, thereby reducing the ability of insect herbivores to feed and digest food [116, 135]. Protease inhibitors, including TrypPIs, act to inhibit the activity of digestive enzymes in the midgut of multiple rice pests, thereby impeding insect development [7, 26].

HIPVs encompass terpenoids, green leaf volatiles (GLVs), aromatic compounds (such as MeSA, methyl benzoate (MeBA), and indole), and others [161, 162]. HIPVs influence the behavior and performance of insect herbivores and their natural enemies, including predators and parasitoids [161]. Notably, different volatile compounds exert varying effects on the same insect (herbivore, parasitoid, or predator), and the same compound may function differently for different insects. In rice, certain volatiles, such as 2-heptanone, 2-heptanol, linalool, (*Z*)-3-hexenal, and (*Z*)-3-hexen-1-ol, are repellent to BPH [19, 150, 153, 158], whereas (*E*)-2-hexenal and (*Z*)-3-hexen-1-ol attract females of the WBPH and LF [157, 163]. MeBA demonstrates strong direct toxicity and repellent effects on *S. frugiperda* [164]. Additionally, volatiles like linalool, (*E*)-β-caryophyllene, (*E*,*E*)-4,8-dimethyl-1,3,7-nonanetriene (DMNT), MeSA, (*Z*)-3-hexenal, (*E*)-2-hexenal, and (*Z*)-3-hexen-1-ol are attractive to *Anagrus nilaparvatae*, an egg parasitoid of rice planthoppers [115, 153–155]. Some volatiles, including (*Z*)-3-hexene-1-ol, linalool, MeSA, DMNT, and (*E*,*E*,*E*)-4,8,12-trimethyltrideca-1,3,7,11-tetraene (TMTT), serve as attractants for *Apanteles chilonis*, a larval parasitoid of SSB [152]. Moreover, volatiles emitted from rice plants attacked by LF, *Mythimna separata*, or *S. frugiperda* can attract corresponding parasitoids such as *Trathala flavo-orbitalis*, *Itoplectis naranyae*, *Microplitis mediator*, and *Cotesia marginiventris* [165–167]. Interestingly, HIPVs may also function as DAMPs, prompting non-attacked tissues on the same plant and/or neighboring plants to respond more rapidly and robustly to subsequent herbivore attacks. For example, pre-exposing rice plants to indole activates the expression of defense genes (*OsMPK3*, *OsWRKY70*, and JA biosynthesis genes) and enhances the accumulation of JA and JA-Ile, priming rice defenses against *S. frugiperda* larvae [168]. Similarly, pre-exposure to rice volatiles emitted from SSB-infested rice plants enhances the direct and indirect resistance of rice to SSB [154].

## Regulation of rice defenses and susceptibility by insect herbivores

To cope with plant resistance or obtain nutrients from plants, adapted herbivores also secrete a repertoire of effectors or factors to suppress plant defenses or enhance plant susceptibility [4, 6, 7] (Fig. 1).

### Suppressing defense responses

By now, many herbivore effectors that suppress plant defenses have been identified. Like elicitors, herbivore effectors may also be present in oral secretions, excreta (frass or honeydew), and oviposition-associated secretions [1, 2]. In rice, such effectors have also been identified in piercing-sucking insect herbivores, principally in the BPH and SBPH (Table 1). As stated above, the infestation of BPH, SBPH or the green rice leafhopper (*Nephotettix cincticeps*) causes a significant Ca^2+^ influx in rice plants and induces resistance [16, 53, 57]. In response, rice planthoppers and *N. cincticeps* secrete calcium-binding proteins, like NlSEF1 from BPH [16], LsECF1 from SBPH [53], and NcSP84 from *N. cincticeps* [57], into rice tissues during feeding, and they bind free cytoplasmic Ca^2+^, which impairs rice defenses. The DNase II secreted from SBPH salivary glands degrades the extracellular DNA that is released from damaged plant cells in response to herbivore infestation, which prevents the triggering of rice defenses, including H_2_O_2_ bursts [54]. Overexpressing an odorant-binding protein (NlugOBP11) or heat shock 70 kDa protein cognate 3 (NlHSC70-3), which is secreted from BPH salivary glands in *N. benthamiana* leaves, inhibits the production of SA and the bacterial elicitor flg22-induced ROS [51, 52]. Moreover, effectors secreted from BPH salivary glands, such as the endo-β-1,4-glucanase (NlEG1) [48] and salivary protein 7 (NlSP7) [50], degrade celluloses in rice cell walls (the former effector) and mediate the metabolism of rice tricin (the latter effector). The C-terminal subunit of SBPH Vg (LsVgC) targets a rice TF, OsWRKY71, to inhibit H_2_O_2_-mediated rice defense [55]. LsSP1, which can be secreted into rice plants during SBPH feeding, interacts with rice papain-like cysteine proteases to impair SA biosynthesis and subsequent SA-mediated rice defenses [56]. Thus far, no effectors derived from rice-chewing insects have been identified. Recently, Xue et al. [58] reported that mechanically wounding plants and applying oral secretions from antibiotic-treated SSB larvae to damaged areas induced higher levels of JA-mediated rice defenses compared to levels induced by mechanically wounding plants and treating them with oral secretions from non-antibiotic-treated SSB larvae; these results suggest that SSB larvae may exploit orally secreted microbes to avoid hyperactivating JA-mediated rice defenses, thereby facilitating the fitness of SSB larvae [58].

Effectors may also inhibit plant defenses by inducing the expression of genes that encode negative regulators of immune signaling [169, 170]. In rice, several genes, such as *OsLRR-RLK2* [13], the simple extracellular LRR gene *OsI-BAK1* [171], *OsMAPK20-5* [100], the E3 ubiquitin ligase gene *OsJMJ715* [18], *OsNPR1* [124], the 13-lipoxygenase gene *Osr9-LOX1* [172], and the histone H3K9 methyltransferase gene *OsSDG703* [173], have been reported to negatively regulate defense-related signaling pathways and plant resistance to insect herbivores; moreover, the expression of these genes can be induced by herbivore infestation, suggesting that insect herbivores may suppress rice defenses by secreting effectors to regulate the expression of these genes. However, such of these effectors have not been identified in rice insect herbivores.

### Inducing susceptible responses

To establish a population on a plant species, insect herbivores need to obtain nutrients from plants that satisfy their growth, development, and fecundity requirements. For this purpose, insect herbivores may also secrete effectors or factors to regulate the expression of the genes that allow the herbivore to reproduce, i.e., genes that induce plant-susceptible responses [169, 174]. In rice, herbivore infestation has been well documented to increase the levels of some compounds that are crucial for herbivore survival, growth, and development. For example, BPH infestation significantly increases the levels of some sugars (sucrose and glucose) and amino acids (alanine, Val, Ile, cysteine, proline, phenylalanine, glutamate, glutamine, histidine, and asparagine) in rice varieties that are susceptible to BPH but elicits few changes in resistant rice plants [22, 23]. Sucrose and some amino acids (aspartate, glutamine, and alanine) can serve as feeding stimulants of BPH [175, 176]. Lack of cysteine, histidine, and methionine in an artificial diet can reduce the survival rate of BPH nymphs and prolong their developmental duration [175]. Recent research has found that BPH or SSB infestation induces the biosynthesis of serotonin, an indole alkaloid in rice, and this compound contributes to the growth and development of BPH and SSB [177, 178]. These results demonstrate that rice insect herbivores may promote their fitness by inducing rice to become more susceptible to them. However, so far, no such effectors or factors have been reported.

## Conclusions and perspectives

The extensive study of molecular interactions between plants and insect herbivores started at the beginning of this century. During the past 20 years or more, many mechanisms underlying plant–herbivore molecular interactions, such as those affecting both the production of herbivore-induced plant defenses and how insect herbivores regulate plant defense, have been found. These molecular mechanisms promote the establishment of a plant–insect interaction model. However, many aspects of the model still need to be clarified. First, although many herbivore elicitors and effectors have been identified, how plants detect these herbivore signals and then activate defenses in response, such as initiating early signaling events and activating phytohormone signaling, remains unclear. Moreover, how insect herbivores suppress plant defenses by secreting effectors, and what the targets of the effectors are are also unknown. Second, it has been observed that infestation by different insect herbivores induces different plant defenses [3, 37, 174]. However, which early, middle, and late components or pathways in plant defense responses shape the specificity of these responses is still unknown. Third, similar to pathogens, insect herbivores may also induce plant-susceptible responses, as stated above. However, how insect herbivores affect this process remains largely unknown. Fourth, only a limited number of defensive compounds in plants, including rice, have been confirmed to both directly and indirectly affect the growth, survival, development, and fecundity of insect herbivores. Hence, it is important to identify plant defensive compounds and elucidate their mechanisms of action. Moreover, exploring how insects perceive and respond to plant defensive compounds will also be essential. Fifth, in the field, plants generally face infestation by multiple insect herbivores, together with the natural enemies of insect herbivores and other organisms, simultaneously or successively. However, how plants and insect herbivores cope with this complex situation remains to be elucidated. Finally, abiotic factors (such as light, temperature, water, and soil fertility) can influence plant defense [179–181]. Moreover, insect herbivores may exploit the mechanisms by which plants resist these factors and suppress plant defenses for their own benefit [129]. How abiotic factors regulate the molecular interaction between plants and insect herbivores deserves future study. Expanding studies to these unexplored aspects would deepen the understanding of plant-insect interactions.

A comprehensive understanding of plant-insect molecular interactions in rice should inform the development of new control methods for insect pests, such as agents that induce plants to resist insects [9, 182], attractants or repellents for insect pests and their natural enemies [8, 9], and new varieties with broad-spectrum and durable resistance to pests or with resistance to both biotic and abiotic stresses [183, 184]. Moreover, the elucidation of plant-insect molecular interactions will lay a solid foundation for developing measures that can synergistically control multiple pests at the same time. These new measures, techniques, and/or strategies will promote the sustainable management of crop pests.

## Data Availability

This article comprehensively summarizes the latest literature in the field. All information and references cited in this review paper are duly acknowledged and properly cited within the text and in the reference list. Data sharing is not applicable to this article.
